# Developing a 3D intestinal epithelium model for livestock species

**DOI:** 10.1007/s00441-018-2924-9

**Published:** 2018-09-26

**Authors:** Hayley Derricott, Lisa Luu, Wai Yee Fong, Catherine S. Hartley, Luke J. Johnston, Stuart D. Armstrong, Nadine Randle, Carrie A. Duckworth, Barry J. Campbell, Jonathan M. Wastling, Janine L. Coombes

**Affiliations:** 1 Department of Infection Biology, Institute of Infection and Global Health and School of Veterinary Science, Faculty of Health and Life Sciences, University of Liverpool, Merseyside, UK; 2 Institute of Integrative Biology, University of Liverpool, Liverpool, UK; 3 Department of Cellular and Molecular Physiology, Institute of Translational Medicine, University of Liverpool, Liverpool, L69 3GE UK; 4 Department of Gastroenterology, Institute of Translational Medicine, University of Liverpool, Liverpool, UK; 5 Faculty of Natural Sciences, Keele University, Keele, Staffordshire ST5 5BG UK

**Keywords:** Organoid, *Toxoplasma*, *Salmonella*, Bovine, Porcine

## Abstract

The in vitro 3D culture of intestinal epithelium is a valuable resource in the study of its function. Organoid culture exploits stem cells’ ability to regenerate and produce differentiated epithelium. Intestinal organoid models from rodent or human tissue are widely available whereas large animal models are not. Livestock enteric and zoonotic diseases elicit significant morbidity and mortality in animal and human populations. Therefore, livestock species-specific models may offer novel insights into host-pathogen interactions and disease responses. Bovine and porcine jejunum were obtained from an abattoir and their intestinal crypts isolated, suspended in Matrigel, cultured, cryopreserved and resuscitated. ‘Rounding’ of crypts occurred followed by budding and then enlargement of the organoids. Epithelial cells were characterised using immunofluorescent staining and confocal microscopy. Organoids were successfully infected with *Toxoplasma gondii* or *Salmonella typhimurium*. This 3D organoid model offers a long-term, renewable resource for investigating species-specific intestinal infections with a variety of pathogens.

## Introduction

The intestinal epithelium performs numerous critical functions key to an organism’s growth and survival. It must permit the absorption of nutrients, act as a barrier to the risks posed by the external environment, respond to pathogenic insults, secrete mucins and lubricating mucous (Peterson and Artis 2014; Barker 2014), produce hormones (Gunawardene et al. 2011) and maintain a constant turnover of cells (Barker 2014). Small intestinal epithelium is organised into crypt-villus domains where the villus epithelium comprises absorptive enterocytes, hormone-producing enteroendocrine cells and goblet cells that produce and secrete mucus. Stem cells that replenish the heterogeneous differentiated epithelial cell types and Paneth cells with antimicrobial functions are found residing at the base of the crypts (Heo and Clevers 2015; Peterson and Artis 2014). Studying events that occur in the gastrointestinal epithelium in vivo is hampered by the sheer scale of the tissue involved. For example, typical enteric infections involve small foci that are easily lost in the background noise of uninfected tissue (Coombes et al. 2013). In vitro cultures have been utilised to gain important insights into the structure and function of intestinal epithelium though these studies have generally been based on primary cells, tissue explants or monolayers. Primary cells cultured as monolayers and epithelial cell lines such as Caco-2 may not recapitulate accurately the phenotype of the epithelium, be it due to a lack of heterogeneity or due to de-differentiation over time (Briske-Anderson et al. 1997; Sambuy et al. 2005; Rusu et al. 2005; Booth et al. 1999). Whilst explant cultures retain their complex architecture and cellular heterogeneity, their use is limited to the short term; foetal tissue may remain viable for 2–3 weeks whereas adult tissue degenerates within 48 h (Dedhia et al. 2016; Chopra et al. 2010). More recently, developments in cell culture techniques have enabled great advances in the maintenance of intestinal epithelial cells in vitro.

The culture of intestinal epithelium into 3D structures, typically termed organoid culture, has proved to be a valuable resource in the study of its function. Organoid culture is based on the ability of intestinal epithelial stem cells (IESC), located at the base of the crypt, to perpetually divide and produce a fully differentiated, polarised epithelium. Through a process of renewal, migration and differentiation, intestinal epithelium undergoes complete regeneration every 4–5 days (Barker 2014). Clever’s group were the first to describe the presence of fast-cycling intestinal stem cells that expressed high levels of mouse leucine-rich repeat-containing G protein-coupled receptor 5 (Lgr5) (Barker et al. 2007). This group subsequently cultured differentiated crypt-villus structures from Lgr5+ stem cells and in doing so, opened wide the possibility of studying the morphological and physiological properties of intestinal epithelium in an in vitro setting (Sato et al. 2009). Thus far, in vitro models of intestinal epithelium have attempted to use mouse, rat or human tissue as translational models of human epithelial function. This is generally due to the availability and ease of use of rodents in the research setting. Large animal models have been mostly overlooked, in part because of the prohibitive costs of maintaining these animals in a research environment. However, from a veterinary, public health and agricultural perspective, 3D epithelial cultures from livestock animals could prove to be highly beneficial, potentially providing insight into development, morphology, cellular differentiation, host-pathogen interactions and drug treatments.

Animal health and welfare is an important issue in international food security and supply. Agricultural economics relies on healthy herds; the continuing presence of zoonotic diseases impacts heavily upon animal and human health. Toxoplasmosis is a widespread global disease, being present in every country and chronically in 30–50% of humans. Infections can be spread via several routes but the ingestion of undercooked meat presents an important route of entry into the host GI tract (Clough and Frickel 2017; Dubey and Jones 2008; Torgerson and Mastroiacovo 2013). Agricultural economies in developing countries have the potential to be devastated by the effects, on both humans and animals, of enteric disease (Tomley and Shirley 2009). Reported seroprevalence of *T. gondii* in cattle and pigs in 2000 was 0–92% and 0–97% respectively (Tenter et al. 2000). Since that time, there have been significant reductions in the prevalence of *Toxoplasma* in pig meat due to indoor farming; however, the increase in organic and free-range farms is seeing this prevalence rise once more (Wallander et al. 2016). The gastrointestinal tract represents one of the primary routes of entry for pathogens and, correspondingly, diarrhoeal disease forms a major global health burden (Ahs et al. 2010). *Salmonella spp*. are a common contaminant of poultry and livestock, typically being carried asymptomatically in the animal’s gastrointestinal tract (Doyle and Erickson 2006). Along with other GI pathogens, they contribute to foodborne diarrhoeal disease, of which an estimated 76 million cases occur annually in the US alone (Ahs et al. 2010; Buzby and Roberts 2009; Doyle and Erickson 2006). It is also stated that animal diseases are one of the most important barriers in the international trade that is so critical for low- and middle-income countries (King et al. 2006). The welfare, financial and public health implications of livestock responses to enteric pathogens are reflected in a real necessity to develop reliable, reproducible models of epithelial morphology and function. The well-documented rise in antibiotic and anthelmintic resistance leads to limited options for the control of enteric infections and there is an urgent need to develop new and more effective drugs, vaccines and adjuvants. Knowledge of infection events would enable the potential to reveal novel strategies for blocking pathogen invasion, neutralising toxins or stimulating the immune response.

An experimental ideal for enteric infection research would involve species-appropriate in vitro gut models that support pathogen replication and can be manipulated experimentally. Matching microbe to host is important in order to accurately understand the pathological processes that occur. Mouse models of disease are not always translatable to large animals or humans due to extensive differences in anatomy and physiology (Ziegler et al. 2016; Young 2009) and may require genetic manipulation in order to achieve the disease state. This, combined with increasing pressure on researchers to replace, refine and reduce animal experimentation (3R’s), means being able to use tissue from adult animals destined for the food chain provides an attractive proposition and a ready, ethically sound supply of tissue.

Little is known about the differentiated cell types present in bovine intestinal epithelium and whether all the cell types described in murine/human epithelium are also present in the bovine epithelium. Morphological studies of bovine intestine have been published in the context of food production and consumption, e.g., sausage casings (Wijnker et al. 2008) and the effects of animal diet on mucosal architecture (Montanholi et al. 2013). However, these studies have not used immunofluorescent staining to identify specific cell types. A recent paper by Powell and Behnke described the successful culture of organoids from small companion and large farm animals. Whilst this study used PCR to demonstrate the presence of the stem cell marker LGR5 and proliferative zones in organoids, it did not investigate the presence of differentiated terminal epithelial cell types (Powell and Behnke 2017). Organoids derived from porcine intestine have been produced though have not been utilised to the same extent as murine organoids. A study published in 2013 established and characterised porcine organoids, confirming the presence of a marker of crypt proliferation, SOX9, along with enteroendocrine cells, goblet cells and absorptive enterocytes (Gonzalez et al. 2013). It is not stated categorically that the animals used in the study were bred specifically for research, though this is implied.

In contrast to using cell lines, monolayers and explants, organoids could provide the ability to manipulate and specify what comes into contact with the luminal surface of the differentiated epithelium. Producing livestock organoid models would provide the foundations to advance knowledge of enteric disease and host-pathogen interactions. In this study, we set out to culture porcine organoids and develop novel 3D organoid models of bovine intestinal epithelium to complement the murine organoids that are already being utilised in our facility. We developed a protocol that produces organoids from tissue that would otherwise be disposed of, from animals destined for the food chain. The 3D organoids we have cultured display differentiated epithelial cells and can be maintained in the long term, cryopreserved and resuscitated with little loss of viability. In addition, these livestock epithelial organoids are susceptible to infection with enteric pathogens that are of global significance to both human and animal health.

## Materials and methods

### Bovine crypt isolation and organoid culture

**Fig. 1:**
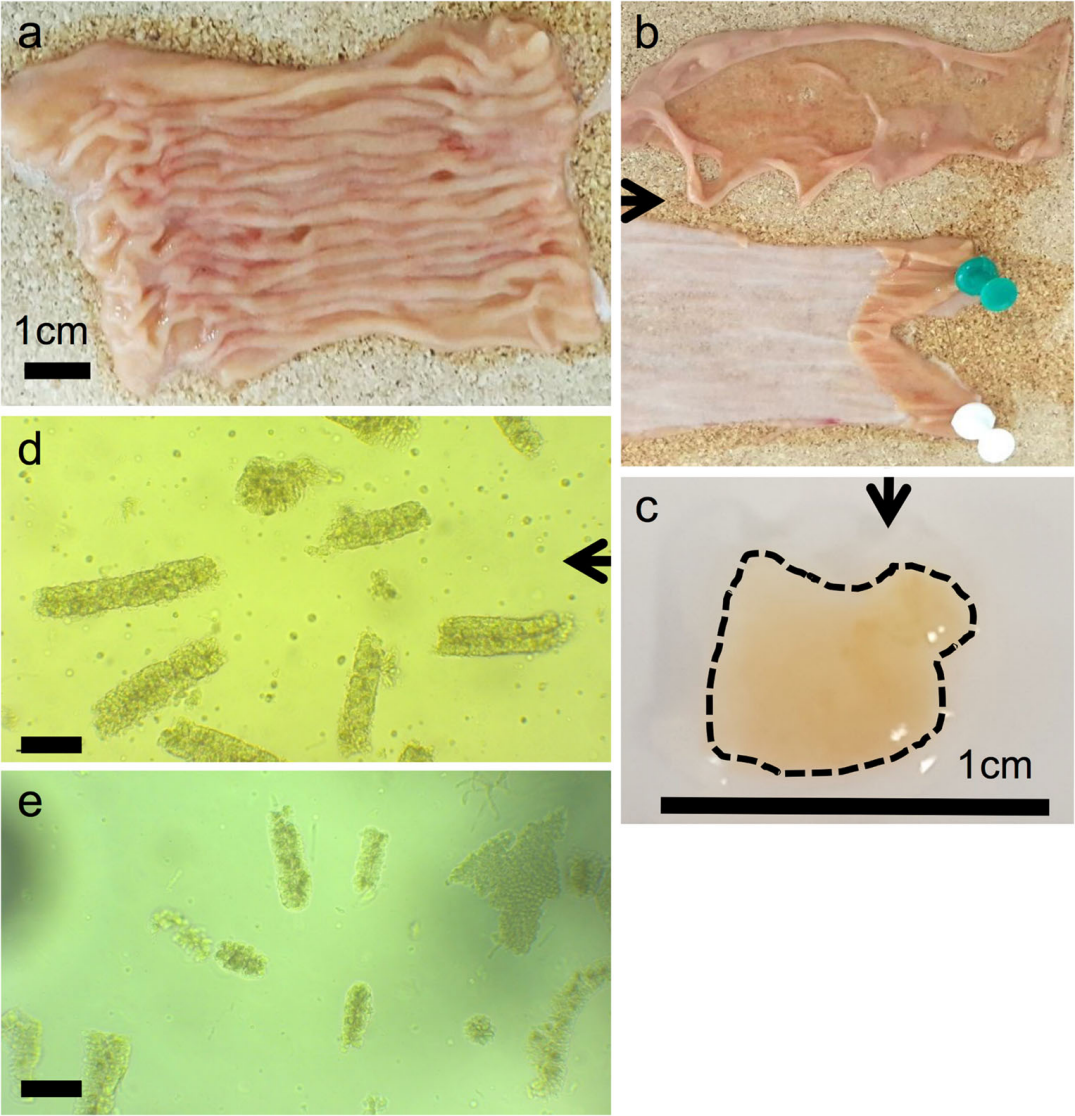
The process of intestinal crypt isolation. (a) A section of jejunum is taken from either a pig or cow slaughtered for food production and cut longitudinally. (b) The mucosal and muscle layers (top) are separated from the serosa (bottom) by scraping with a microscope slide. (c) Approximately 50–60 tissue fragments of 5 mm^2^ are produced. Following 30-min incubation in EDTA, a series of crypt fractions are produced, the best of which is centrifuged and embedded in Matrigel. (d) Isolated bovine crypts. (e) Isolated porcine crypts. Original magnification × 100, panels (d) and (e) scale bar represents 20 μm

Sections of proximal jejunum (approximately 10 cm in length and ~ 1-m distal to the duodenum) from adult cows between 20 and 30 months of age were obtained within 30 min of slaughter from a Food Standards Agency (FSA)-approved slaughterhouse within 10 miles of the laboratory (Fig. 1a). Tissue was acquired following the procedures and restrictions described in the Derogations from Animal By-Product controls under Regulation (EC) 1069/2009 and Commission Regulation (EU) 142/2011 (DEFRA 2011). It was cut and opened longitudinally before being placed and transported in 25-ml ice-cold Hank’s Balanced Salt Solution (HBSS) supplemented with 100 μg/ml Primocin (InvivoGen, CA, USA). Jejunal sections were gently scraped to remove the mucous layer and villi before being washed in PBS at room temperature (RT) until the supernatant appeared clear. The mucosal layer of the intestine was removed by scraping with a glass microscope slide (Fig. 1b). Tissue was cut into approximately 50–60 5-mm^2^ pieces (Fig. 1c) before a 30-min incubation (with intermittent swirling) in 20-ml ice-cold 0.8-mM PBS/EDTA solution (Lonza). At the end of EDTA incubation, the tube was shaken for 10 s to dislodge villus tissue and the contents poured into a Petri dish. Intestinal pieces were then placed into 20-ml Dulbecco’s PBS (DPBS) without Ca^2+^/Mg^2+^, shaken for a further 10 s and poured into a Petri dish. The pour-off formed fraction 1, which was examined under a microscope to determine the proportion of villus and crypt tissue present. Tissue was transferred to 20 ml of fresh DPBS and the shaking step repeated up to four times to produce a selection of epithelial fractions. The fraction with the highest ratio of crypts to villi was selected for the next stage of the process (Fig. 1d). Based on the density of the crypts in the selected fraction, we calculated that 0.5–1 ml of crypt suspension was required for each well of organoids to be cultured. The required volume of crypt suspension was centrifuged at 50*g* for 3 min at 4 °C, the supernatant aspirated then the crypt pellet resuspended in a pre-prepared mix of 70% Matrigel (Corning), 30% bovine medium (1:1 mixture of IntestiCult (Stem Cell Technologies, Cambridge, UK)) and Wnt3a-conditioned medium (WntCM) supplemented with 1 μg/ml human recombinant R-spondin, 100 ng/ml murine Noggin, 100 ng/ml murine epidermal growth factor (EGF; Peprotech, London, UK), 1.5-μM CHIR99021, 5-μM Y27632, 5-μM SB202190, 250-nM A8301 (Tocris, Bristol, UK) and 100 μg/ml Primocin (InvivoGen, Toulouse, France) and 30 μl applied on coverslips in a pre-warmed 48-well plate. Following 30 min Matrigel polymerisation time at 37 °C, 200 μl of bovine medium at RT was added to each well. Cultures were incubated at 37 °C, 5% CO_2_. Medium was changed every 3–4 days until the organoids required passage (~ 7–10 days).

### Porcine crypt isolation and organoid culture

As with the bovine tissue, sections of proximal jejunum (approximately 10 cm in length) from adult pigs were obtained from an FSA approved slaughterhouse within 10 miles of the laboratory. Tissue was acquired following the procedures and restrictions described in the Derogations from Animal By-Product controls under Regulation (EC) 1069/2009 and Commission Regulation (EU) 142/2011 (DEFRA 2011). Tissue was prepared for crypt isolation as described previously for bovine tissue (Fig. 1). We calculated that 0.5–1 ml of crypt suspension was required for each well of organoids to be cultured. The required volume of crypt suspension was centrifuged at 125*g* for 7 min at 4 °C, the supernatant aspirated then the crypt pellet resuspended in a pre-prepared mix of 70% Matrigel (Corning), 30% IntestiCult (Stem Cell Technologies) with 100 μg/ml Primocin (InvivoGen) and 30 μl applied on coverslips in a pre-warmed 48-well plate. Following 30-min Matrigel polymerisation time at 37 °C, 200 μl of IntestiCult at RT was added to each well. Cultures were incubated at 37 °C, 5% CO_2_. Medium was changed every 3–4 days until the organoids required passage (~ 7 days).

### Murine crypt isolation and organoid culture

Mice used in organoid studies were maintained in accordance with the Animals Scientific Procedures Act 1986 (ASPA). All mice used were female, specific-pathogen free C57B1/6J strain, aged between 6 and 12 weeks (Charles River, Margate, United Kingdom) and sacrificed by cervical dislocation. Removal of the distal half of the small intestine was performed in the Biological Specimens Unit (BSU) of the University of Liverpool. Biological samples were transported for further processing in ice-cold PBS. The distal half of the murine small intestine was opened longitudinally and flushed out with ice-cold PBS to remove the luminal contents. Tissue was segmented into ~ 5–10-mm lengths, transferred into ice-cold dissociation solution (15-ml PBS, 900-μl 0.5-M EDTA) and incubated for 5 min at room temperature. Tissues were transferred into 15-ml ice-cold PBS in 50-ml tube and shaken vigorously for ~ 15 s to release luminal mucus and villi into the supernatant (fraction). The liquid content of the tube was transferred into a Petri dish, the tissue re-incubated in the dissociation solution for a further 5 min and shaken in PBS. This was repeated three times in order to generate a total of four crypt fractions. The fractions were checked by light microscopy for crypt enrichment and the fraction containing the highest ratio of crypts to villi was selected. The supernatant was filtered through a 70-μm strainer and quantified by counting the number of crypts in a 20-μl aliquot. Crypts were pelleted by centrifugation at 300*g* for 10 min at 4 °C and resuspended in a pre-prepared mix of 70% Matrigel (Corning), 30% IntestiCult (Stem Cell Technologies) before applying 30 μl on coverslips in a pre-warmed 48-well plate. Following 30-min Matrigel polymerisation time at 37 °C, 200 μl of IntestiCult at RT was added to each well. Cultures were incubated at 37 °C, 5% CO_2_ and medium changed every 3–4 days until the organoids required passage.

### Organoid passage

Organoids remained in culture for up to 10 days until they appeared to be ‘crowded’ in the Matrigel and this was when passage was deemed necessary. Medium was removed and 500-μl room temperature DPBS added to the wells. Organoid-containing Matrigel was resuspended in the DPBS and transferred to a centrifuge tube. All of the organoids suspended in the Matrigel were fragmented to separate the crypts/villi by gentle pipetting in the tube before centrifugation. The organoid fragment suspension was centrifuged at 50*g* for 3 min at 4 °C (bovine) or 300*g* for 10 min at 4 °C (porcine and murine). Supernatant was aspirated, taking care to avoid the organoid pellet that was then resuspended in the 70% Matrigel, 30% IntestiCult/bovine medium mix described previously. Passaged organoids in Matrigel were plated onto pre-warmed coverslips at 30 μl per well. Following a polymerisation period of 30 min, 200 μl of IntestiCult/bovine medium at RT was added to the wells.

### Cryopreservation and resuscitation of organoids

At day 6 or 7 of culture, medium was removed from wells and 500-μl room temperature DPBS added. Organoid-containing Matrigel was resuspended in the DPBS and transferred to a centrifuge tube. The organoids were fragmented by gentle pipetting in the tube before centrifugation. The organoid fragment suspension was centrifuged at 50*g* for 3 min at 4 °C (bovine) or 300*g* for 10 min at 4 °C (porcine). Supernatant was removed and organoid pellets resuspended in CryoStor® (Stem Cell Technologies) using a volume of 1-ml CryoStor® per three wells organoids. Vials were frozen at – 80 °C for 24 h in a Nalgene® Mr Frosty freezing container (Thermo Fisher Scientific, Cheshire, UK) before being transferred to liquid nitrogen for long-term storage.

For resuscitation, vials were removed from liquid nitrogen and defrosted in a waterbath at 37 °C until the freezing medium became liquid but not warm. The solution in the vial was transferred to a 15-ml tube with 5-ml IntestiCult added. Suspensions were centrifuged at 50*g* for 3 min at 4 °C (bovine) or 300*g* for 10 min at 4 °C (porcine). Organoid pellets were then resuspended in Matrigel and covered with medium as per the passage process.

### Establishing and maintaining *Toxoplasma gondii* culture in vero cells

Vero cells were cultured in 5-ml medium (high glucose Dulbecco’s Modified Eagle’s Medium (DMEM)) supplemented with 5% foetal bovine serum (FBS) and 1% penicillin/streptomycin (P/S, 100 IU/ml penicillin, 100 μg/ml streptomycin; all Sigma–Aldrich, Dorset, UK) and incubated in a T25 flask at 37 °C, 5% CO_2_. Confluent monolayers were trypsinised and subsequently sub-cultured by seeding at 4 × 10^5^ cells/flask. After overnight incubation of freshly seeded vero cells, culture medium was changed, a defrosted cryovial of *T. gondii* RH tachyzoites was added and incubated at 37 °C, 5% CO_2_. Cultures were examined by light microscopy using an inverted microscope to determine the presence of tachyzoites and the percentage of cells infected. Once approximately 75% of the vero cell monolayer was infected and signs of parasite egress were observed, the culture was considered ready to either passage or harvest. Passage of parasites to continue culture involved the transfer of the supernatant from infected flasks to a T25 containing freshly seeded vero cells.

### Harvesting *Toxoplasma gondii* for organoid infection

Adhered vero cells infected with tachyzoites were released from the bottom of the flask by scraping. The cell/parasite suspension was passed through a blunt ended needle several times to mechanically disrupt the cells and release intracellular parasites. To purify tachyzoites, the suspension was passed through PD-10 desalting columns (GE HealthCare Life Sciences, Buckinghamshire, UK) as per manufacturers’ instructions and a haemocytometer used to quantify the number of parasites. Parasites were added to organoid cultures at a quantity of 1 × 10^6^ per well. The required volume of parasite suspension was centrifuged at 2000 rpm for 10 min at RT before being resuspended in IntestiCult complete medium (for porcine cultures) or bovine medium.

### *Salmonella typhimurium* culture for organoid infections

*Salmonella enterica* subspecies enterica serovar Typhimurium strain 4/74 carrying a chromosomal rpsM::gfp fusion linked to the cat resistance gene (JH3599) was used for the pilot infections of bovine organoids. The 4/74 strain is the prototrophic parent to *S. Typhimurium* SL1344 (Hoiseth and Stocker 1981) and was isolated from a calf bowel (Rankin and Taylor 1966). Strain JH3599 was constructed by P22 phage transduction from a SL1344 strain carrying a chromosomal rpsM::gfp fusion (JH3016) (Hautefort et al. 2003) into wild-type 4/74 background, as previously described (Lemire et al. 2011). Strain JH3599 was grown overnight in 5-ml LB-Lennox broth (10 g/l tryptone, 5 g/l yeast extract, 5 g/l NaCl) supplemented with chloramphenicol (25 μg/ml) at 37 °C with shaking at 225 rpm. In order to maximise invasion of mammalian cells by inducing expression of the *Salmonella* pathogenicity island 1 (SPI-1), 300 μl of the overnight culture was sub-cultured into 10-ml LB-Miller broth (10 g/l tryptone, 5 g/l yeast extract, 5 g/l NaCl) in a 150-ml loose-capped glass Erlenmeyer flask and incubated at 37 °C for 3.5 h with shaking at 225 rpm (Antonio Ibarra et al. 2010).

### Infection of bovine, porcine and murine organoids with *Toxoplasma gondii* or *Salmonella typhimurium*

Infection of organoids was carried out by disrupting the 3D organoid structure to expose the luminal surface and incubating the fragments with parasites. As per the previously described organoid passage protocol, medium was removed and 500-μl DPBS added to the wells. Matrigel was resuspended in DPBS by gentle pipetting and transferred to a centrifuge tube. The organoid fragment suspension was centrifuged at 50*g* for 3 min at 4 °C (bovine) or 300*g* for 10 min at 4 °C (porcine). Supernatant was aspirated, taking care to avoid the organoid pellet. *T gondii* RH infections were carried out using 1 × 10^6^ parasites suspended in 5-μl medium per well. The organoid pellet was resuspended in the appropriate volume of parasite suspension and incubated for 1 h at 37 °C, 5% CO_2_. After 1 h, the organoid/parasite suspension was mixed with the required volume of Matrigel/medium (described previously) and plated at 30 μl onto coverslips in a pre-warmed 48-well plate. After allowing the Matrigel to polymerise for 30 min, 200-μl IntestiCult or bovine medium was added to the porcine or bovine organoids respectively. Infected wells were incubated for 24 h at 37 °C, 5% CO_2_ before being fixed for 1 h at RT with 4% paraformaldehyde (PFA). Pilot infection of porcine organoids with GFP *S. typhimurium* 4/74 involved application of 1 × 10^6^ bacteria suspended in IntestiCult to wells of organoids that remained embedded in Matrigel. Organoids were incubated with bacteria at 37 °C, 5% CO_2_ for 1 h. Pilot infection of murine and bovine cultures was carried out by initial fragmentation and centrifugation of organoids as per the passage process. Fragments were subsequently incubated with 1 × 10^7^ bacteria per well at 37 °C, 5% CO_2_ then embedded in Matrigel and allowed polymerising for 45 min. Following incubation, organoids (murine, porcine and bovine) were fixed for 1 h at RT with 4% PFA.

### Immunofluorescent staining of organoids

A standard protocol for immunofluorescent staining was followed. Briefly, organoids were fixed in 4% PFA for 1 h at RT. Wells were washed for 2.5 h with wash buffer (PBS with 1% donkey serum and 0.1% Triton X-100) on a rocker. Non-specific binding was blocked and organoids were permeabilised using PBS with 10% donkey serum and 1% Triton X-100 (blocking buffer) for 4 h at RT on a rocker. Primary rabbit (R) or mouse (M) antibodies (R anti-chromogranin A, R anti-mucin 2, R anti-e-cadherin and M anti-SAG1 (surface antigen 1)) were diluted 1:200 in blocking buffer; 200 μl was applied to each well and incubated at 4 °C overnight on a rocker followed by three washes with washing buffer. Donkey anti-rabbit FITC or TRITC or donkey anti-mouse Alexa Fluor (AF) 488 secondary antibodies, diluted 1:200 in wash buffer, with rhodamine or AF647-conjugated phalloidin (F-actin detection) diluted 1:250 were applied to the appropriate wells and incubated for a further 2 h at RT. Wells were washed with PBS for 2 h, with the addition of DAPI nuclear stain (Invitrogen, MA, USA) for the final 10 min. Coverslips were mounted onto slides with Hydromount medium (National Diagnostics, Nottingham, UK).

### Confocal imaging of stained organoids

Immunofluorescent stained coverslip images were captured using Zen Black software with a Zeiss LSM 880 multiphoton confocal upright microscope (Zeiss, Cambridge, UK). Laser lines Diode 405-30 (405 nm), argon (488 nm), DPSS-5610 (561 nm) and HeNe633 (633 nm) were used. All confocal images were captured using a W-Plan Apochromat 40x objective with water immersion or W-Plan Apochromat 63x objective with oil immersion.

### Proteomic analysis of bovine organoids

Analysis was performed on five wells of densely packed organoids using three biological replicates. Organoid tissue was prepared for proteomic analysis as per our laboratory’s standard protocol. Briefly, five wells of organoids in Matrigel were resuspended in 500-μl RT DPBS (without Ca^2+^/Mg^2+^). The suspension was centrifuged at 2000 rpm for 10 min at RT, supernatant aspirated and the pellet resuspended in PBS. The washing step was repeated a further two times in order to remove the Matrigel from the suspension. After the final wash the supernatant was aspirated and the organoid pellet stored at − 20 °C for later analysis.

Buffer (50-mM ammonium bicarbonate (ambic) with 0.1% *w*/*v* Rapigest (Waters, Hertforshire, UK)) was added to washed organoid samples then sonicated on ice using a sonicating waterbath (Jencons, Leicestershire, UK) for 3 × 10 min. Samples were heated at 80 °C for 10 min before protein content was determined using the Coomassie Plus (Bradford) assay kit (Pierce Scientific). Protein content was normalised between samples using 50-mM ambic. Incubation of samples in 3-mM dithiothreitol (Sigma Aldrich, Dorset, UK) for 10 min at 60 °C reduced proteins that were then alkylated, in the dark, with 9-mM iodoacetimde (Sigma Aldrich) at room temperature for 30 min. Proteomic grade trypsin (Sigma Aldrich) was added at a protein:trypsin ratio of 50:1 and samples incubated at 37 °C overnight. Rapigest was removed followed by the addition of TFA at a final concentration of 1% *v*/*v* and a 2-h incubation at 37 °C. Peptide samples were centrifuged at 12,000*g* for 60 min (4 °C) to remove precipitated Rapigest.

### NanoLC MS ESI MS/MS analysis

Peptides were analysed by on-line nanoflow LC using the Ultimate 3000 nano system (Dionex/Thermo Fisher Scientific). Samples were loaded onto a trap column (Acclaim PepMap 100, 2 cm × 75 μm inner diameter, C18, 3 μm, 100 Å) at 9 μl/min with an aqueous solution containing 0.1% (*v*/*v*) TFA and 2% (*v*/*v*) acetonitrile. After 3 min, the trap column was set in-line an analytical column (Easy-Spray PepMap® RSLC 50 cm × 75 μm inner diameter, C18, 2 μm, 100 Å) fused to a silica nano-electrospray emitter (Dionex). The column was operated at a constant temperature of 35 °C and the LC system coupled to a Q-Exactive mass spectrometer (Thermo Fisher Scientific). Chromatography was performed with a buffer system consisting of 0.1% formic acid (buffer A) and 80% acetonitrile in 0.1% formic acid (buffer B). The peptides were separated by a linear gradient of 3.8–50% buffer B over 90 min at a flow rate of 300 nl/min. The Q-Exactive was operated in data-dependent mode with survey scans acquired at a resolution of 70,000 at *m*/*z* 200. Up to the top 10 most abundant isotope patterns with charge states + 2 to + 5 from the survey scan were selected with an isolation window of 2.0Th and fragmented by higher energy collisional dissociation with normalised collision energies of 30. The maximum ion injection times for the survey scan and the MS/MS scans were 250 and 50 ms, respectively and the ion target value was set to 1E6 for survey scans and 1E5 for the MS/MS scans. MS/MS events were acquired at a resolution of 17,500. Repetitive sequencing of peptides was minimised through dynamic exclusion of the sequenced peptides for 20 s.

Thermo RAW files were imported into Progenesis LC–MS (version 4.1, Nonlinear Dynamics). Runs were time aligned using default settings and using an auto selected run as reference. Peaks were picked by the software and filtered to include only peaks with a charge state of between + 2 and + 6. Peptide intensities were normalised against the reference run by Progenesis LC–MS and these intensities were used to highlight differences in protein expression between control and treated samples with supporting statistical analysis (ANOVA *p* values) calculated by the Progenesis LC–MS software. Spectral data were transformed to .mgf files with Progenesis LC–MS and exported for peptide identification using the Mascot (version 2.3.02, Matrix Science) search engine. Tandem MS data were searched against the bovine predicted proteome (Uniprot release 2013_09). Mascot search parameters were as follows: precursor mass tolerance set to 10 ppm and fragment mass tolerance set to 0.05 Da. One missed tryptic cleavage was permitted. Carbamidomethylation (cysteine) was set as a fixed modification and oxidation (methionine) set as a variable modification. Mascot search results were further processed using the machine learning algorithm Percolator. The false discovery rate was < 1%. Individual ion scores > 13 indicated identity or extensive homology (*p* < 0.05). Protein identification results were imported into Progenesis LC–MS as .xml files.

### Consent for use of tissue

Bovine and porcine tissue was acquired from FSA approved abattoirs following the procedures and restrictions described in the Derogations from Animal By-Product controls under Regulation (EC) 1069/2009 and Commission Regulation (EU) 142/2011 (DEFRA 2011). These regulations permit the transport and use of animal tissue (not for human consumption) in research and education. Murine intestine used within this study was obtained from animals maintained with accordance to the Animals (Scientific Procedures Act 1986 (ASPA).

## Results

### Viable organoids can be made from adult livestock animals

There is a dearth of livestock-specific intestinal epithelial models that accurately recapitulate in vivo morphology. We set out to produce porcine and bovine 3D organoids using jejunal tissue obtained from animals slaughtered for food production purposes.

Initially, processing of both porcine and bovine tissue utilised the established protocol devised for the isolation and culture of murine crypts. However, neither species produced viable organoids with isolated crypts deteriorating overnight and dying within 24 h. Both the bovine and porcine crypt isolation protocols required modifications from the murine protocol in order to preserve the integrity of the crypts. Fractions were not passed through a cell strainer as the crypts were too large (Fig. 1d, e) and would not have been separated from the villus debris. In addition, the centrifugation step was altered so that the crypts did not become dissociated as this affected their viability. Whereas murine crypt isolates are centrifuged at 150*g*–300*g* for 10 min (Mahé et al. 2013; Wilson et al. 2015), we found that the larger porcine and bovine crypts broke up if processed at these speeds and thus we reduced the centrifugation step to 125*g*/7 min for porcine crypt isolates and 50*g*/3 min for bovine.

**Fig. 2:**
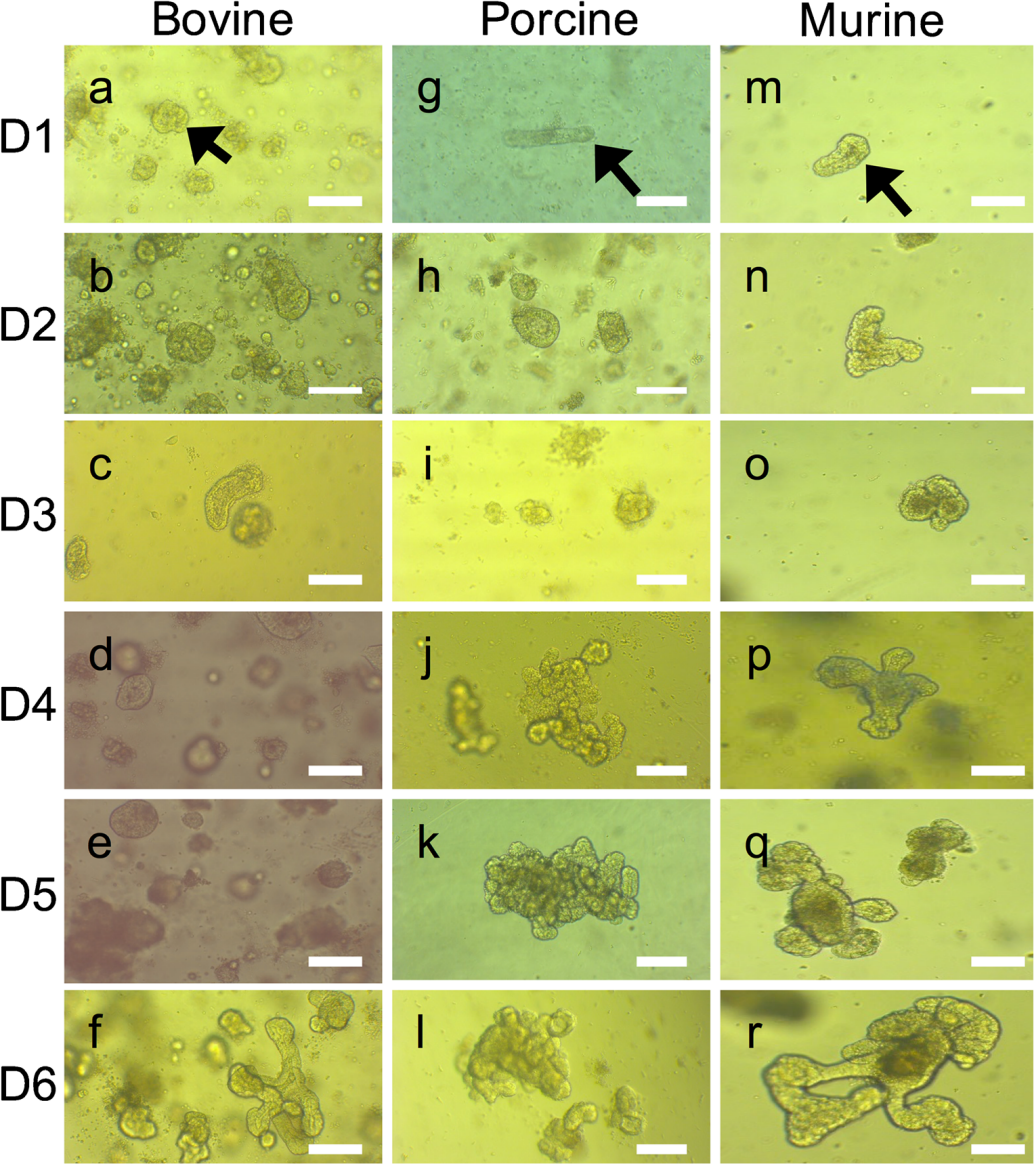
Comparison of bovine (a–f), porcine (g–l) and murine (m–r) early organoid morphology from day 1 to day 6. Intestinal crypts (arrows in a, g and m) can be seen to start rounding off with budding observed at day 6 in bovine organoids (f) and day 3–4 in porcine and murine organoids (j, o and p). Original magnification × 100, scale bar represents 40 μm

The development of organoids from crypt isolation to first passage resembles that seen in murine organoid cultures, which is already well established in our laboratory (Fig. 2m–r). At day 1, post-isolation rounding of the crypts can be observed. Both the bovine (Fig. 2a–f) and porcine (Fig. 2g–l) cultures have more cellular debris in the Matrigel, which is related to the fact that the crypt fractions cannot be sieved before plating. In comparison to murine organoids, the porcine cultures appear to develop quite slowly in the first 3 days of culture (Fig. 2g–l) but are of comparable size by day 6. However, bovine organoids (Fig. 2a–f) appear to have a slight lag on their development, with the first passage generally being required approximately 10 days post set-up compared to 7 days for porcine (Fig. 2g–l) and murine (Fig. 2m–r) cultures.

IntestiCult, a proprietary defined serum-free medium designed specifically for the establishment and culture of murine organoids, was used as a base medium for all organoid cultures. Porcine organoids grew and survived in IntestiCult alone; however, we were unable to maintain bovine organoids in this medium; they were non-viable within 24 h of embedding in Matrigel. The medium used for bovine organoid cultures required manipulation to existing medium recipes and incorporation of supplementary growth factors and inhibitors. Referring to the literature relating to bovine primary cell and murine and porcine organoid cultures (Khalil et al. 2016; Mahé et al. 2013; Fuller et al. 2012; Wilson et al. 2015; Sato et al. 2009; Dedhia et al. 2016; Fatehullah et al. 2016; Date and Sato 2015; Gonzalez et al. 2013), we added WntCM, growth factors R-spondin (Wnt agonist), Noggin (TGFβ superfamily inactivator) and EGF (to enhance epithelial proliferation) along with inhibitors A83-01 (TGFβ type 1 receptor), SB202190 (MAP 3K), Y27632 (ROCK) and CHIR99021 (GSK3), which provided the necessary components to establish bovine organoids. To test that IntestiCult was required as the base medium, in one bovine culture, IntestiCult was replaced with DMEM (our murine organoid medium), which resulted in the isolated crypts dying within 24 h (data not shown).

Porcine cultures remain viable for up to 13 passages, which equates to approximately 3 months in culture. After this time, organoids begin to dissociate and viability is reduced, although epithelial differentiation still occurs (data not shown). Bovine cultures show more variability in their longevity; some cultures remained viable for four passages whereas others have been passaged 12 times and are still viable and healthy. There is also considerable variability in the time elapsed between passages; sometimes passage was required within 4 days, at others passage was carried out after 10 days. Therefore, even with lower passage numbers, some bovine organoids potentially have the capability to last for more than 12 weeks.

### Organoids can be cryopreserved and resuscitated whilst maintaining viability

Understanding that it may not be feasible for researchers to access abattoir-generated material and in order to produce a reliable resource that presents an option for long-term cultures, it is desirable to be able to cryopreserve and resuscitate organoids.

**Fig. 3:**
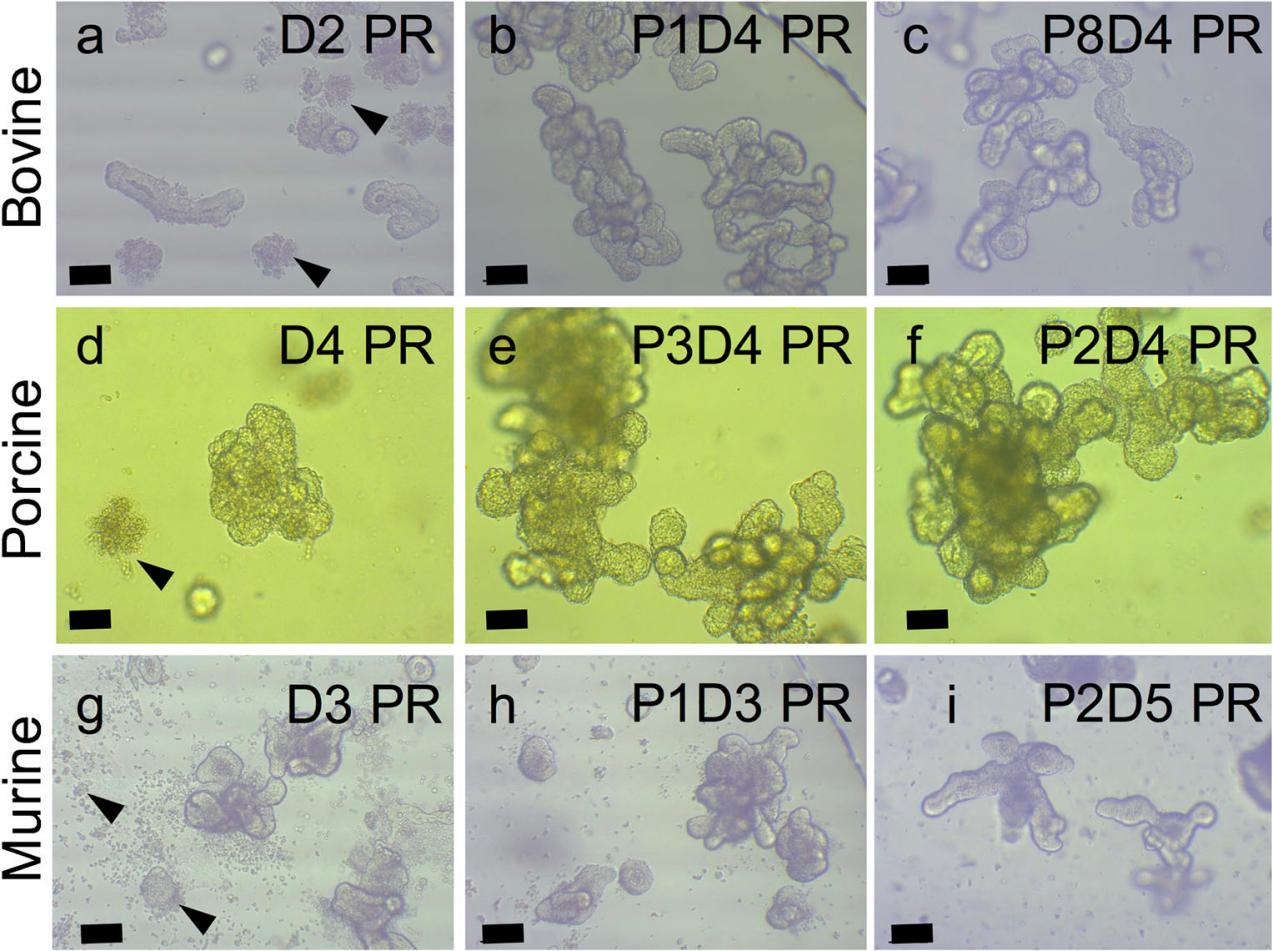
Comparison of bovine (a–c), porcine (d–f) and murine (g–i) organoid morphology after cryopreservation then resuscitation into Matrigel. In the first 2–4 days, post-resuscitation (PR) small patches of non-viable cellular debris can be observed (arrows in a, d and g) though these are reduced/removed by subsequent passages. Original magnification × 100, a–c and g–i, scale bar represents 200 μm, d–f, scale bar represents 100 μm

Murine, porcine and bovine organoids were all successfully cryopreserved in CryoStor® and were resuscitated with a viability of approximately 75–80% (Fig. 3). In the days immediately following resuscitation, patches of non-viable cellular debris are visible. Subsequent passages remove the debris allowing the viable organoids to be embedded in Matrigel.

### Porcine and bovine organoids demonstrate a differentiated epithelial phenotype

In order to recapitulate the morphology of the in vivo intestinal epithelium, it was necessary to produce well-differentiated 3D organoids that displayed the range of cell types that would be expected in vivo*.* Immunofluorescent staining of porcine and bovine organoids demonstrated that differentiation of epithelial cell types had occurred.

**Fig. 4:**
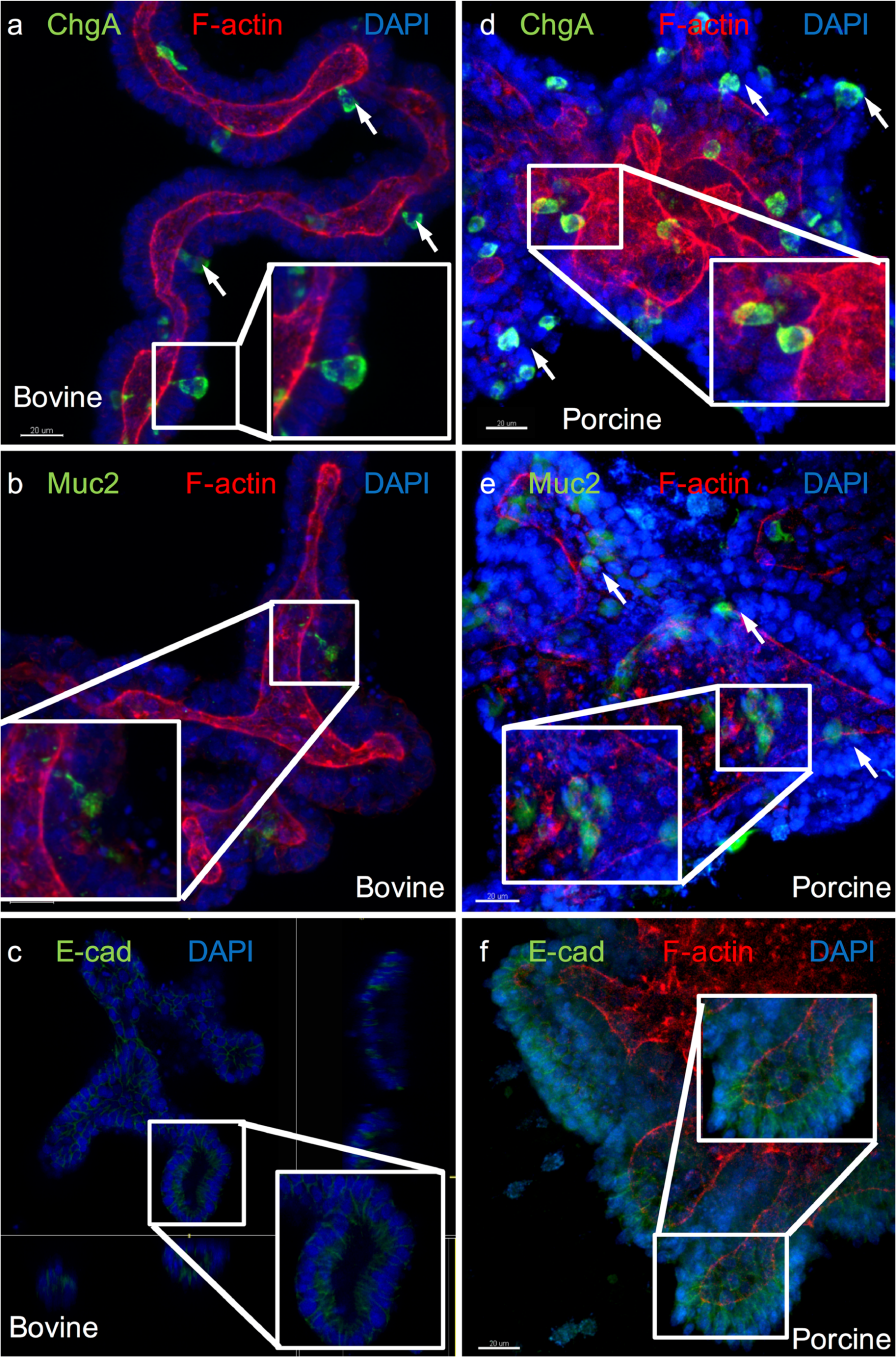
Immunofluorescent staining of bovine (a–c) and porcine (d–f) intestinal organoids to show evidence of epithelial differentiation. (a and d) Rabbit anti-chromogranin A (ChgA—enteroendocrine cells, white arrows and insets shows examples), (b and e) Rabbit anti-mucin 2 (Muc2—goblet cells, white arrows, insets), (c and f) Expression of rabbit anti-E-cadherin (E-cad—epithelial adherens junctions, insets. Panels a–f) Green, FITC; red, rhodamine (f-actin); blue, DAPI. Original magnification × 400, scale bar represents 20 μm

Positive chromogranin A, mucin 2 and E-cadherin staining was observed, indicating that enteroendocrine cells, goblet cells and adherens junctions, respectively, were present. In addition, positive f-actin staining with differential staining on the luminal surface of the organoids implies that the epithelium was polarised as it would be in vivo (Fig. 4).

### Differentiated intestinal and epithelial proteins are detectable in bovine organoids

In order to confirm that the organoid structures cultured accurately recapitulated in vivo epithelium, mass spectrometry label-free quantitative proteomics was used to ascertain global protein expression profiles of bovine organoid cultures.

Using an exclusion criteria of > 2 peptides per protein identified, a total of 2134 proteins were detected in bovine organoids. A total of 522 uncharacterised proteins were listed, 493 of which displayed a gene name. Cross-reference of known murine intestinal epithelial Uniprot ID numbers/gene names with bovine uncharacterised proteins that had gene names produced a list of proteins that were either intestine- or epithelium-specific (Tables 1 or 2). Functions of epithelial-specific proteins included the development and maintenance of cell junctions and cell polarisation (Table 1). Functions of intestinal-specific proteins included enterocyte and stem cell markers, mucus components and villus morphogenesis (Table 2).

**Table 1:** Selected epithelial-related proteins identified by label-free mass spectrometry in bovine intestinal epithelial organoids

Uniprot ID	Gene name	Protein name	Function
F1MEG3	LAMA1	Laminin subunit alpha 1	Basal lamina protein
F1N2X7	AFDN	Afadin	Cell junction development
Q3T0L5	EPCAM	Epithelial cell adhesion molecule	Tight junction formation
Q3B7N4	CLDN7	Claudin-7	
Q765P1	CLDN2	Claudin-2	
F1MBN2	TJP2	Tight junction protein 2, ZO-2	Tight junction protein
F1N2D3	TJP1	Tight junction protein 1, ZO-1	
F1N789	VCL	Vinculin	Focal adhesion formation
F1MFC2	DSG2	Desmoglein-2	Hemidesmosome/desmosome proteins
E1BKT9	DSP	Desmoplakin	
E1BFQ6	ITGA6	Integrin subunit alpha 6	
E1B864	ITGB4	Integrin beta	
E1BF59	PLEC	Plectin	
F1MU12	KRT8	Keratin, type II cytoskeletal 8	Intermediate filament component
Q29S21	KRT7	Keratin, type II cytoskeletal 7	
A6QQQ9	KRT20	Keratin, type II cytoskeletal 20	
P08728	KRT19	Keratin, type II cytoskeletal 19	
P53712	ITGB1	Integrin beta-1	Maintenance of polarisation

The presence of epithelial-specific proteins in the organoids are suggestive that the tissue is differentiating into an epithelium and is maintaining its structural integrity (*n* = 3)

**Table 2:** Selected intestinal-related proteins identified by label-free mass spectrometry in bovine intestinal epithelial organoids

Uniprot ID	Gene name	Protein name	Function
A6H742	PLS1	Plastin 1	Actin bundling in brush border
Q5E9Z3	VIL1	Villin 1	Enterocyte marker
Q56JX9	FABP2	Fatty acid-binding protein, intestinal	Enterocyte marker
P31976	EZR	Ezrin	Epithelial organisation and villus morphogenesis
A6H7H6	CDH17	Cadherin 17	Intestinal cell-cell adhesion
Q3T0I2	CTSH	Pro-cathepsin	Intestinal homeostasis
F1MVJ8	OLFM4	Olfactomedin-4	Intestinal stem cell marker
E1BAQ3	MUC13	Mucin 13	Mucus component
F1N3J3	AGR2	Anterior gradient 2	
G5E5Q6	TFF3	Trefoil factor 3	
F1MB08	ENO1	Alpha-enolase	
Q5E9B7	CLIC1	Chloride intracellular channel protein 1	
E1BBS6	QSOX2	Sulphhydryl oxidase	Pluripotency

Intestine-specific proteins identified using label-free mass spectrometry are suggestive that tissue is displaying an intestinal phenotype and is differentiating into terminal cell types (*n* = 3)

### Porcine and bovine organoids are susceptible to infection by *Toxoplasma gondii*

Porcine and bovine organoids were developed in order to produce a physiologically relevant and species-specific model to be used for the in vitro study of enteric infections. Infection with *T. gondii* parasites was chosen due to its ability to infect the cell of most warm-blooded mammals and its prevalence in the global population.

**Fig. 5:**
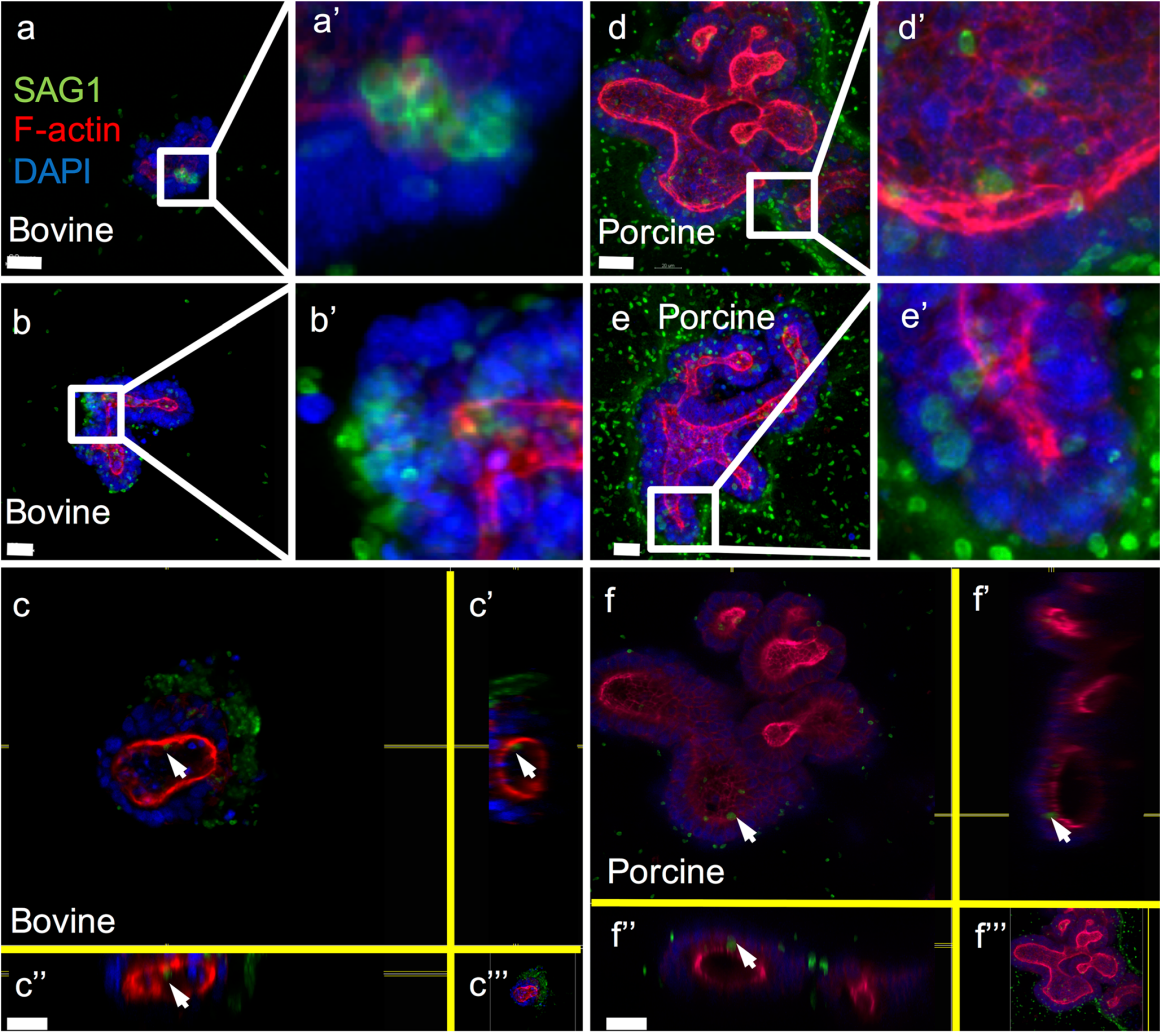
Bovine (a–c) and porcine (d–f) intestinal organoids infected with 1 × 10^6^*Toxoplasma gondii* RH parasites. Organoids were incubated with parasites for 1 h at 37 °C, embedded in Matrigel and incubated for a further 24 h at 37 °C. Parasite surface antigen 1 (SAG1; Alexa Fluor 488—green), f-actin (rhodamine—red) and nuclei (DAPI—blue) are shown. Panels a and d and zooms (panels a′ and d′) illustrate intraluminal parasites. Panels b and e and zooms (panels b′ and e′) show parasite rosettes indicating that replication is occurring (c, c′, c″, f, f′ and f″). Image sections show intracellular parasites (white arrows). Panels a, b, d and e scale bar represents 20 μm, panels c and f, 30 μm. Original magnification × 400

Incubation of porcine and bovine organoids with 1 × 10^6^*T. gondii* RH parasites for 1 h with subsequent culture in Matrigel for 24 h produced detectable foci of infection (Fig. 5).

### Porcine and bovine organoids are susceptible to infection by *Salmonella typhimurium*

Physiologically relevant models of intestinal epithelium could be utilised in the study of early post-infection events that occur with a variety of pathogens overcoming the difficulties associated with locating foci of infection in an in vivo model.

**Fig. 6:**
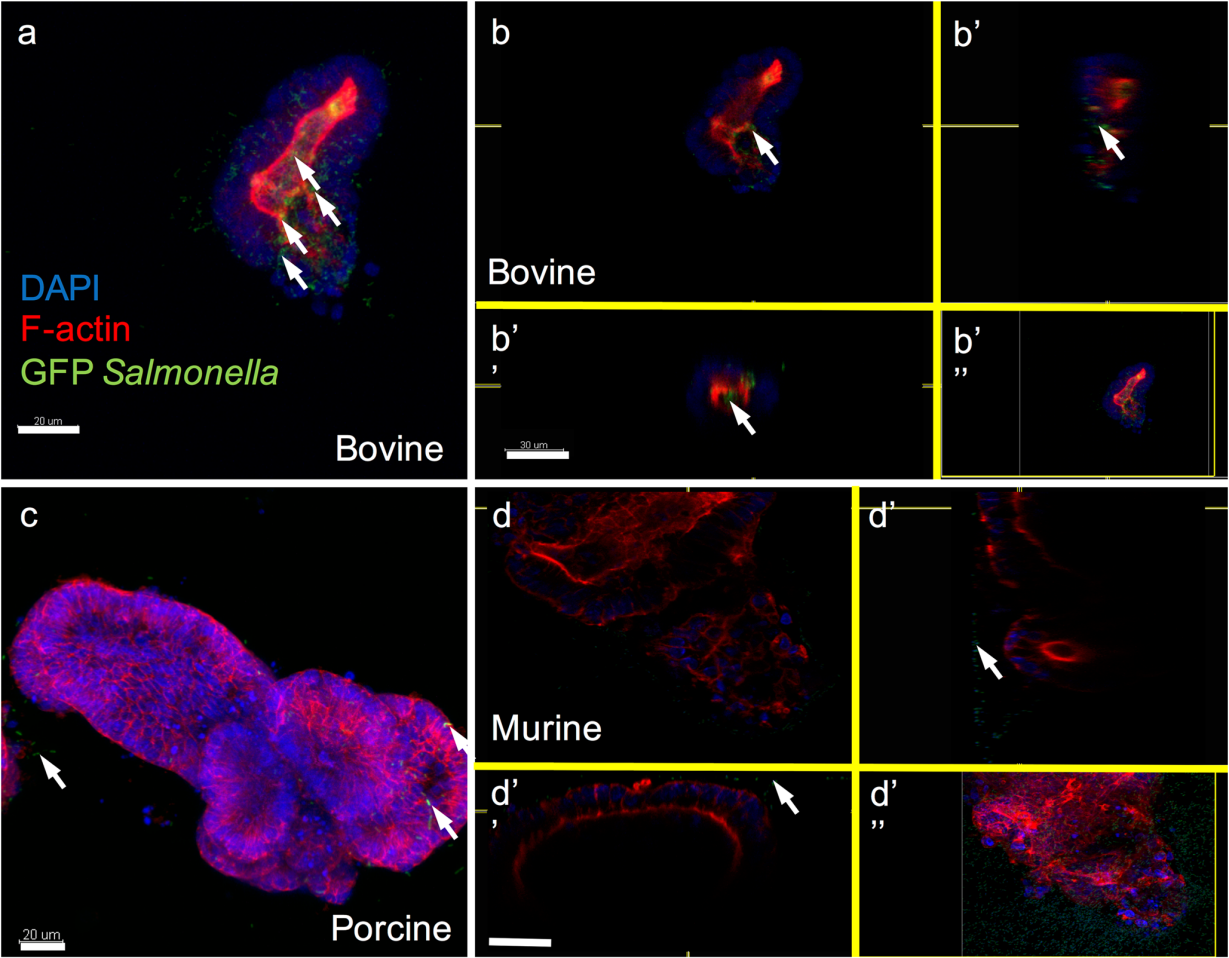
Pilot culture of livestock organoids with GFP-tagged *Salmonella typhimurium* 4/74 (a, b, b′, b″ and b‴). Luminal bacteria (green) can be seen in bovine organoids cultured for 45 min with 1 × 10^7^ bacteria (white arrows). (c) Porcine organoids cultured with 1 × 10^6^ bacteria for 1 h. Bacteria (green) have migrated through the Matrigel and are visible on the periphery of the intact organoids (white arrows), though no foci of infection are detectable (d, d′, d″ and d‴). Bacteria (green) can be seen at the periphery of murine organoids cultured for 45 min with 1 × 10^7^ bacteria (white arrows) but no foci of infection are visible. F-actin is stained with rhodamine phalloidin (red) and nuclei with DAPI (blue). Panels a, b and d, scale bar represents 30 μm, original magnification × 630. Panel c, scale bar represents 20 μm, original magnification × 400

*S. typhimurium* 4/74 strain was chosen for infection as it is an enteric bacterium that is highly virulent in cattle after oral challenge (Villarreal-Ramos et al. 2000). There is no published evidence that mice are susceptible to challenge with this bacteria thus mouse organoids were utilised as a control. Culturing fragmented bovine organoids with 1 × 10^7^ GFP-tagged *S. typhimurium* 4/74 for 45 min resulted in the presence of luminal bacteria (Fig. 6a, b). The pilot culture of intact porcine organoids with 1 × 10^6^ GFP-tagged *S. typhimurium* 4/74 for 1 h demonstrated that the bacteria were able to migrate through Matrigel in order to approach the organoids (Fig. 6c) though no foci of infection were observed. The control culture of murine organoids incubated with *S. typhimurium* 4/74 did not produce any visible foci of infection (Fig. 6d).

## Discussion

The utility of murine and human organoid cultures in the study of human development, physiology, disease and drug toxicity has been well cited (Date and Sato 2015; Zachos et al. 2015; Mahé et al. 2013; Fatehullah et al. 2016). Organoids present a valuable resource that permits the in vitro study of a functional epithelium that additionally retains genetic homogeneity (Date and Sato 2015). However, the potential importance of organoid cultures in veterinary health and zoonotic disease research has been largely overlooked. This study has successfully established porcine and bovine intestinal organoids and demonstrated that they are susceptible to infection by bacteria and parasites. By modifying techniques and protocols that are well established in our laboratory, we were able to consistently isolate crypts and produce 3D cultures. Aside from the prohibitive costs of rearing and keeping large animals in an animal unit, concerted efforts to refine, reduce or replace animals in the research setting mean that in vivo experiments using livestock are generally considered unfeasible and rarely approved (Reynolds et al. 2008; Roberts et al. 2009). We have shown that utilising tissue from adult animals slaughtered for food (that would otherwise be unused) provides a feasible, accessible, ethically sound and continuous source of material. This then enables the study of livestock intestinal epithelium in vitro and overcomes the translational issues of species mismatch in studies of enteric physiology, disease and zoonoses.

Immunofluorescent characterisation of epithelial cell types in both the bovine and porcine organoids proves that the isolated crypts were capable of regeneration and differentiation. Identification of positive staining for chromogranin A and mucin-2 imply that cellular differentiation is producing functional villus cells. Furthermore, the presence of the adherens junction protein e-cadherin and apically expressed f-actin are strongly suggestive that the in vitro epithelium model is polarised as it would be in vivo. It has also been suggested that *Salmonella* more efficiently invade polarised rather than non-polarised epithelium (Lorkowski et al. 2014), again implying that the model mimics the in vivo situation. Detection by mass spectrometry of intestinal- and epithelial-specific proteins further confirms that organoids represent an appropriate in vitro model for investigating the anatomy and physiology of the small intestine of livestock species.

The organoids described here have been shown to be viable for at least 3 months whilst retaining their capacity to differentiate. Qualitative assessment of the density of crypts embedded in Matrigel also revealed that a high density of crypts plated in the initial cultures produced more successful organoid transformation. Whilst Powell and Behnke’s 2017 study produced bovine and porcine cultures capable of undergoing up to 49 passages, the organoid morphology they described is suggestive of a less differentiated structure with proliferative areas but little budding observed. This may be, in part, due to the medium used for the culture of the organoids. Powell and Behnke showed that conditioned medium from the L-WRN cell line was sufficient to support the growth of organoids (Powell and Behnke 2017). Whilst we also utilised the growth factors Wnt3a, R-spondin and Noggin, we additionally supplemented our organoid medium with epidermal growth factor (EGF) and various inhibitors that may have influenced the survival and proliferative capacity of the isolated crypts by reducing apoptosis in the cultures. ROCK inhibitor Y27632 is well cited in the literature (Khalil et al. 2016; Mahé et al. 2013; Fuller et al. 2012; Wilson et al. 2015; Sato et al. 2009; Dedhia et al. 2016; Fatehullah et al. 2016) as an inhibitor of dissociation-induced apoptosis (anoikis); A83-01 is a TGFβ receptor 1 inhibitor, therefore, a promoter of epithelial growth, SB202190 a p38 MAPK inhibitor essential for organoid growth (Date and Sato 2015) and CHIR99021 a GSK inhibitor reported to enhance cell survival in porcine organoids (Gonzalez et al. 2013). The supplements were added to the base medium IntestiCult, which is designed for use in murine and human intestinal organoid cultures. We found that IntestiCult alone was sufficient to support the development and culture of porcine organoids whereas the medium needed considerable supplementation to sustain bovine organoids. The advantage of producing organoids with a differentiated phenotype for use in infection studies is the presence of the range of epithelial cell types that would be required for response to a pathogenic insult.

Whilst porcine organoids have been previously produced and characterised by Gonzalez et al. (2013), the aims of the study differed from those described here. They took a translational perspective, aiming to utilise porcine organoids as a physiologically relevant model for human tissue. Whilst this is, no doubt, of interest to the field of medicine, it is worth the reminder that enteric diseases of livestock are fundamental contributors to both animal and human health (Ahs et al. 2010). The use of 3D organoids from livestock species as a model for zoonotic pathogens is a novel concept. The ability to cryopreserve and resuscitate viable organoids further enhances their attractiveness as a renewable and long-term investigative tool. Making these resources available to other establishments provides the tools necessary to enable livestock enteric research.

To prove that the organoids could act as a model for enteric infections, we carried out pilot infections of porcine and bovine cultures with *S. typhimurium* 4/74 and *T. gondii* RH. *S. typhimurium* 4/74 strain is a bovine isolate that is highly virulent after oral challenge and thus was considered suitable for use, particularly with bovine organoids. The protozoan parasite *T. gondii* is capable of infecting virtually any warm-blooded mammal and so presents an ideal pathogen to optimise these novel infection protocols. There are mouse models of salmonellosis that demonstrate susceptibility to infection by the bacteria (Swearingen et al. 2012) so it is interesting to note that incubation of murine organoids with *S. typhimurium* 4/74 did not result in infection, a fact that emphasises the vital requirement of species-specific models of enteric infection. Though we have shown that organoids are susceptible to infection by *T. gondii* and *S. typhimurium* and that *S. typhimurium* will migrate through Matrigel to get to the organoids, there are limitations to the described protocol. During the infection process, organoids were fragmented in order to expose the luminal surface of the epithelium to the parasite/bacteria during incubation. Unfortunately, there is no guarantee that this is the route of entry that the pathogens would use and thus, we cannot definitively say that the infections are an exact physiological replicate of the in vivo situation. In order to achieve this, one would have to use microinjection into the lumen of the closed conformation organoids. Microinjection of *Salmonella typhimurium* SL1344 into organoids derived from human induced pluripotent stem cells has been achieved (Forbester et al. 2015) though the clear aim of this study was the interaction of bacteria with human cells, not animal. Moreover, additional difficulties are faced when attempting to microinject parasites because of the size of the organisms. *T. gondii* parasites are approximately 6 μm by 2 μm and so require a correspondingly large-sized pipette tip for injection. Optimisation of this technique would provide the means to introduce *T. gondii* into the lumen of the organoids and furthermore, it would be possible to use live cell imaging to track the parasites during an infection event. A robust microinjection protocol could also be extended to other parasites of human/veterinary health importance for example *Cryptosporidium parvum* and *Neospora caninum.*

## Conclusion

This study produced renewable, sustainable, differentiated 3D organoids for porcine and bovine species that can be cryopreserved and resuscitated without loss of viability. They can also be infected with *S. typhimurium* and *T. gondii*. The work presented here demonstrates that organoids can be cultured, preserved and resuscitated to investigate species that would otherwise be unfeasible. By using tissue from non-research animals, we negated the logistical, ethical and financial difficulties associated with large animal work and sourced a potentially continuous supply of material. This work addresses the stipulations laid out by the 3R’s to reduce and find alternatives to laboratory experimentation using animals. The protocol we developed could be easily manipulated to extend its scope to other livestock/farm animals. We aim to make the organoids available as a resource to the wider academic community, which would bypass the requirement to have access to abattoir material. Finally, the range of pathogens that can be investigated should not be limited to those described here. There are a great many enteric pathogens of importance to human and veterinary health that could be studied using this in vitro method, which provides a solid base on which to build further studies.
